# Functionalized Reduced Graphene Oxide as a Versatile Tool for Cancer Therapy

**DOI:** 10.3390/ijms22062989

**Published:** 2021-03-15

**Authors:** Banendu Sunder Dash, Gils Jose, Yu-Jen Lu, Jyh-Ping Chen

**Affiliations:** 1 Department of Chemical and Materials Engineering, Chang Gung University, Kwei-San, Taoyuan 33302, Taiwan; banendusunder@gmail.com (B.S.D.); gilsjose84@gmail.com (G.J.); 2 Department of Neurosurgery, Chang Gung Memorial Hospital, Linkou, Kwei-San, Taoyuan 33305, Taiwan; luyj@cgmh.org.tw; 3 Department of Plastic and Reconstructive Surgery and Craniofacial Research Center, Chang Gung Memorial Hospital, Linkou, Kwei-San, Taoyuan 33305, Taiwan; 4 Research Center for Food and Cosmetic Safety, Research Center for Chinese Herbal Medicine, College of Human Ecology, Chang Gung University of Science and Technology, Taoyuan 33305, Taiwan; 5 Department of Materials Engineering, Ming Chi University of Technology, Tai-Shan, New Taipei City 24301, Taiwan

**Keywords:** reduced graphene oxide, chemotherapy, photothermal therapy, photodynamic therapy, gene therapy, immunotherapy

## Abstract

Cancer is one of the deadliest diseases in human history with extremely poor prognosis. Although many traditional therapeutic modalities—such as surgery, chemotherapy, and radiation therapy—have proved to be successful in inhibiting the growth of tumor cells, their side effects may vastly limited the actual benefits and patient acceptance. In this context, a nanomedicine approach for cancer therapy using functionalized nanomaterial has been gaining ground recently. Considering the ability to carry various anticancer drugs and to act as a photothermal agent, the use of carbon-based nanomaterials for cancer therapy has advanced rapidly. Within those nanomaterials, reduced graphene oxide (rGO), a graphene family 2D carbon nanomaterial, emerged as a good candidate for cancer photothermal therapy due to its excellent photothermal conversion in the near infrared range, large specific surface area for drug loading, as well as functional groups for functionalization with molecules such as photosensitizers, siRNA, ligands, etc. By unique design, multifunctional nanosystems could be designed based on rGO, which are endowed with promising temperature/pH-dependent drug/gene delivery abilities for multimodal cancer therapy. This could be further augmented by additional advantages offered by functionalized rGO, such as high biocompatibility, targeted delivery, and enhanced photothermal effects. Herewith, we first provide an overview of the most effective reducing agents for rGO synthesis via chemical reduction. This was followed by in-depth review of application of functionalized rGO in different cancer treatment modalities such as chemotherapy, photothermal therapy and/or photodynamic therapy, gene therapy, chemotherapy/phototherapy, and photothermal/immunotherapy.

## 1. Introduction

Cancer, unrestrained cell growth in the human body, has severely threatened human health worldwide due to its incurability and high death rate [1]. Although reasons for this fatal disease are uncountable, the mechanism of cancer development is associated with the failure of a body’s normal control mechanism, which results in the abnormal proliferation of new cells [2]. Owing to the severity of this disease, researchers and medical professionals have made huge contributions in advancing various treatment modalities—including surgery, chemotherapy, and radiation therapy—for saving human life [3,4]. Although conventional drug delivery systems and treatment approaches have provided some treatment efficacy, its effectiveness is limited by various factors, including multi-drug resistance, rapid metabolism and elimination of drugs, non-specific cytotoxicity, etc. [5,6,7].

Considering the drawbacks of conventional therapies, introduction of nanomaterials in biomedical research has provided a revolutionary application of biomaterials in cancer therapy [8]. Indeed, due to their unique properties, nanomaterials have gained increasing attention for new and innovative use in biomedical research, particularly as nanocarriers for delivery of therapeutic drugs in cancer therapy [9]. To date, a wide variety of nanocarriers—including liposomes, micelles, peptides, and inorganic particles—are being explored as nanovehicles for delivery of cancer therapeutics [10,11,12,13,14,15,16,17,18]. Among these, carbon-based nanomaterials in the graphene family have gained particular attention due to their effectiveness and versatility for cancer treatment [19,20,21,22]. The carbon-based nanomaterials have essential structural and surface features for loading and pH-sensitive release of aromatic anticancer drugs [23]. Considering certain limitations associated with other materials, these nanomaterials attracted tremendous attention for delivery of cancer therapeutics not only due to their unique physico-chemical properties such as high surface area for drug loading [24], but also due to preferred biological properties such as endosomal escape after intracellular uptake for gene delivery and gene therapy [25,26].

The lateral dimensions and thickness of graphene family nanomaterials, such as graphene oxide (GO), reduced graphene oxide (rGO), graphene quantum dots, and graphene nanoribbons can be fine-tuned from original two-dimensional (2D) structure into zero-, one-, or three-dimensional assemblies [27], which provide improved accumulation as drug vehicles and contrast agents at specific target sites [28]. Such unique and tunable features have promised their new applications in drug delivery [29]. Nonetheless, the promise that these nanomaterials have shown in nanomedicine is not only limited to drug delivery, but also in highly sensitive biosensors and high throughput bioassays, as well as scaffolds for tissue engineering [30].

Similar to GO, rGO is a 2D nanomaterial in graphene family with a single-atom-thick layer of sp2 hybridized carbon atoms arranged in a honeycomb lattice structure, which is obtained by reducing GO through chemical, thermal, or electrical methods to eliminate the oxygen-containing functional groups on the surface. Owing to the unique surface property and presence of functional groups, functionalized rGO can accommodate high loading of genes to increase the delivery efficacy of nucleic acid therapeutics in gene therapy. The high surface area also enables the loading of abundant hydrophobic aromatic anticancer drugs for chemotherapy or photosensitizers for photodynamic therapy (PDT) via π–π interaction [31]. Besides being excellent photo-absorbers with high light absorption ability in the near infrared (NIR) range, rGO is associated with pronounced photothermal effect compared with GO, rendering potential applications in cancer photothermal therapy (PTT) [32]. Indeed, with its facile synthesis, high water dispersibility, easy surface functionalization, and good biocompatibility, rGO has emerged as an excellent multifunctional nanomaterial for PTT [33]. After combining this unique characteristic with the high loading capacity of anticancer drugs, rGO reveals itself as a promising nanomaterial for chemo-photothermal therapy [34]. Surface functionalization with multiple therapeutic moieties or conjugation with targeting ligands on rGO surface further permit its use in targeted synergistic cancer therapy such as chemo-phototherapy and photothermal/immunotherapy.

## 2. Preparation of Reduced Graphene Oxide (rGO) by Chemical Reduction

**Figure 1:**
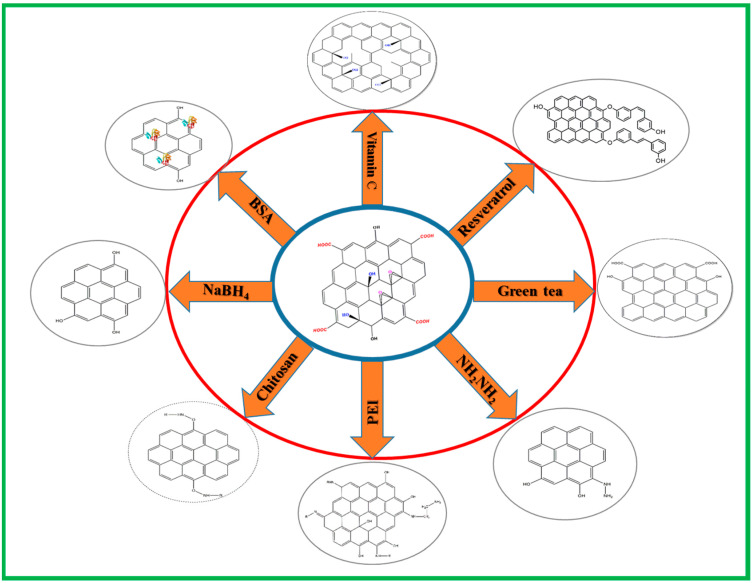
Schematic diagram illustrating the preparation of rGO from GO using different chemical reducing agents. BSA, bovine serum albumin; PEI, polyethyleneimine.

The typical methods for preparation of rGO involve reducing GO by thermal, chemical or electrical methods. Among them, the chemical reduction method that deoxygenates GO with a reducing agent prevails over other non-chemical routes for rGO synthesis, which can produce stable dispersions of rGO with improved quality. Herein, we review the most effective chemical reagents that can act as a direct or indirect reducing agent to convert oxygenated graphene (GO) into rGO (Figure 1). The characteristics of as-produced rGO and its applications are summarized in Table 1.

It should be noted that although different synthesis routes were reported for rGO synthesis, chemical reduction is widely accepted as the most promising method for large-scale production of rGO. However, considering the toxicity of many chemical reducing agents, biomedical application of rGO prefers non-toxic green chemicals for the reduction process, which could provide stability, non-toxicity, and functionality to resulting rGO products. However, as various kinds of rGO synthesized via different routes have been used successfully for cancer therapy, there is still no definite answer regarding which synthesis route could produce the most effective rGO product for application in cancer therapy. 

### 2.1. Vitamin C

Vitamin C (L-hexuronic acid or L-ascorbic acid) is a mild reducing chemical widely used as a reducing agent for GO due to its non-toxicity. It is consider as one of the suitable choices for reducing GOs, not only because it produces highly reduced GO nanosheet suspended in water or in hydrogels at room temperature or mild temperatures, but also because it produces an environmentally friendly by-product, dehydroascorbic acid, after the reaction [35,36]. The chemical reduction of GO by vitamin C has been described in many reports and most of them describing simple mixing of GO with vitamin C using a magnetic stirrer at 60–70 °C and react for 30 min to 2 h. The reduction of GO to rGO is confirmed by color change from brown to black [37,38,39]. A stable suspension of highly reduced GO could be produced using vitamin C in aqueous solution as well as in organic solvent such as dimethylformamide and N-methyl-2-pyrrolidone. Moreover, as vitamin C is composed of carbon, oxygen, and hydrogen, the possibility of introducing heteroatoms to rGO could be avoided.

### 2.2. Hydrazine Hydrate

Hydrazine (N_2_H_4_) or hydrazine hydrate (N_2_H_4_∙H_2_O) is one of the most widely used reductants for rGO synthesis in large scale. Nonetheless, considering the explosive and toxic nature of hydrazine hydrate, the reduction of GO using this reducing agent should be performed with care [40]. Literature describes the reduction process by mixing dispersion of GO with hydrazine hydrate and ammonia solution with a weight ratio of hydrazine to GO at 7:10. After being vigorously shaken or stirred for a few minutes, the reaction was carried out in a water bath at 96 °C for 1 h. Once the reduction is complete, excess hydrazine must be removed by dialysis against 0.5% ammonia solution [41]. In another study, GO was reduced by hydrazine hydrate in the presence of poly(sodium 4-styrenesulfonate) (PSS) to produce stable PSS-coated rGO nanosheet in aqueous dispersion [42]. Treatment of GO (5 mL, 0.5 mg/mL) with hydrazine (0.50 mL, 32.1 mM) at 100 °C for 24 h is another way of reducing GO [43]. There are some other studies that used 80 °C for reaction, where aqueous solution of GO was stirred and sonicated for at least 1 h before reacting with hydrazine hydrate (weight ratio of hydrazine hydrate to GO = 1:1) with continuous stirring and sonication [44]. All studies confirm that hydrazine is a good reducing agent for producing rGO.

### 2.3. Resveratrol

Resveratrol is polyphenol compound used both as a reducing agent and a stabilizer. Resveratrol-guided reduction of GO could provide better biocompatibility, solubility, and selectivity compared to many other reducing agents. The reduction process involves addition of 50 µM resveratrol into GO (1 mg/mL), which was sonicated for 15 min beforehand, and reacted at 40 °C for 1 h. This was continued by cooling and sonication for 15 min, followed by continuous stirring for 1 h at 90 °C. After centrifugation and washing in distilled water, prepared rGO could be recovered from the solution [45].

### 2.4. Chitosan

Chitosan, a biocompatible and biodegradable polysaccharide derived from incomplete deacetylation of chitin, serves as a reducing agent in the synthesis of many nanoparticles [46,47]. Suspension of rGO in aqueous solution has been prepared by chemical reduction of GO at room temperature in the presence of chitosan. To perform the synthesis, a 1:1(*w*/*w*) mixture of GO and chitosan were heated at 37 °C for 72 h under constant stirring. Followed by this, excess chitosan in the solution was removed by centrifugation at 8000 rpm for 1 h and subsequently washed with 2% acetic acid solution. The rGO was dispersed in distilled water by sonication [48,49]. Due to the higher biocompatibility, chitosan-based reduction can enhance the potential biological and medicinal applications of rGO.

### 2.5. Polyethyleneimine (PEI)

Polyethyleneimine (PEI) is a widely used reducing agent and surface modifier in the fabrication of rGO [50], which is a water soluble cationic polymer containing primary, secondary, and tertiary amino groups. To reduce GO, 60 mL of GO (0.1 mg/mL) dispersion and PEI solution was mixed under vigorous stirring at 80 °C for 2 h. The transformation of yellowish-brown to black dispersion indicates the successful transformation of GO to rGO. The mixture was then centrifuged and washed with water for recovery of PEI-rGO [50,51,52]. The incorporation of PEI molecule into GO can act as a source of carbon and produce rGO in a one-step hydrothermal process.

### 2.6. Sodium Borohydride

Sodium borohydride (NaBH_4_) is consider as one of the efficient, nontoxic, noncorrosive, inexpensive reducing agents available for reduction of GO. NaBH_4_ has been frequently used as a reducing agent for aldehydes and ketones to produce alcohols. For synthesizing rGO from GO, a GO suspension (0.5 mg/mL) was mixed with NaBH_4_ as well as CaCl_2_ and stirred for 12 h at room temperature [53]. In another study, the reduction of GO by NaBH_4_ was carried out at different temperatures, showing the highest extent of reduction when the reaction was conducted at 80 °C [54].

### 2.7. Bovine Serum Albumin (BSA)

Bovine serum albumin (BSA) is an affordable protein with high biocompatibility, which can act as a reductant and a stabilizer of GO due to the presence of the amino acid tyrosine (Tyr) within it [55,56]. The reduction of GO was carried out by reacting 1 mg/mL GO solution with 50 mg/mL BSA at 70 °C. After the solution pH was brought up to 12 with 1 M NaOH, the mixture was stirred at 50 °C for 24 h to observe a transition of solution color from light brown (GO) to dark black (rGO) [57]. Excess BSA was removed by centrifugal filtration with a 150 kD molecular-weight-cut-off (MWCO) membrane to obtain purified rGO/BSA hybrids suspended in water and stored at 4 °C [57,58].

### 2.8. Green Tea Polyphenols

Considering the harmful and hazardous natures of many commercial chemical reducing agents, researchers have focused on green methods for the production of rGO by chemical reduction. As a result, green tea extract is considered as a good option considering its easy availability, eco-friendly characteristics, and cheap price. Green tea is rich in polyphenolic compounds, with epigallocatechin gallate (EGCG) making up about 50−60% of total tea polyphenols. To reduce GO, green tea powder (2 g) was added to 100 mL of deionized water and boiled at 100 °C for 20 min, and then filtered. The GO (50 mg) was added to the green tea solution and sonicated for 30 min, followed by reflux at 90 °C under nitrogen atmosphere. After that, the solution was washed with water to remove excess green tea powder [59]. Alternatively, 10 mL green tea extract was added dropwise to 20 mL of GO aqueous suspension (0.5 mg/mL) within 45 min and the mixture was refluxed at 60 °C for 6 h before precipitation of rGO out of the solution [60,61].

## 3. Application of Reduced Graphene Oxide (rGO) in Cancer Therapy

rGO-based nanocomposite has emerged as a promising nanomaterial in nanomedicine. Most recent cytotoxicity studies indicate that surface functionalization of rGO could lead to enhanced biocompatibility as well as increased stability in physiological buffers. Therefore, the use of rGO-based nanomaterials for targeted pH-responsive drug delivery may overcome current challenges and provide new treatment modality in cancer therapy. Nonetheless, other than cytotoxicity study, the distribution and excretion of rGO-based nanomaterials is of paramount importance before clinical translation. The first hurdle that rGO-based cancer therapeutics faces will be the reticuloendothelial system (RES) after intravenous delivery. Overall, nanoparticles with a particle size of ~100 nm are expected to have prolonged circulating half-lives, which should be the preferred size of rGO-based nanocomposites. After escaping the RES, circulating rGO can exit tumor blood vessels and accumulate in cancerous interstitium due to the leaky tumor blood vessels by the enhanced permeation and retention (EPR) effect. For rGO-based nanomaterials to extravasate the vasculature, they should preferably possess neutral or negative charge and be within 10–100 nm in size. Indeed, nanomaterials coated with biocompatible moieties with size smaller than 100 nm are believed to be cleared from the body without noticeable toxicity after systemic administration. Even though the EPR effect may increase accumulation at the tumor site, improving the active targeting ability of rGO-based nanocomposites or using administration route other than intravenous injection should be studied. A systematic study of the biological behavior of injected rGO in vivo—such as stability, biodistribution, secretion, etc.—should be attempted for better clinical use. Furthermore, the functionalization of rGO may be difficult in large scale, which might limit the application of functionalized rGO in cancer therapy from bench to bedside.

**Figure 2:**
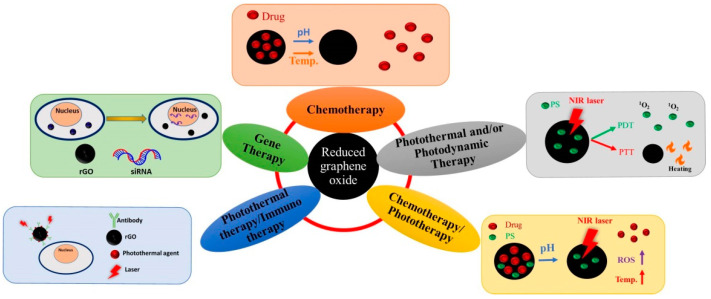
Applications of reduced graphene oxide (rGO) in cancer therapy.

rGO is widely accepted for application in single mode cancer therapy such as chemotherapy, photothermal therapy (PTT), photodynamic therapy (PDT), and gene therapy as well as in dual mode cancer therapy including chemotherapy/phototherapy and photothermal therapy/immunotherapy. A schematic diagram illustrating the mechanisms involved is depicted in Figure 2. In this section, we categorize most up-to-date studies using functionalized rGO-based nanocarriers in cancer therapy into several sections based on the treatment modality involved. A summary of rGO-based nanocarriers, the agents used for functionalizing rGO, cancer cell lines used in the study and the type of study is provided in Table 2.

### 3.1. Chemotherapy

The chemotherapy that cures malignancy by means of chemical drugs has gained worldwide acceptance due to its capability to block cell proliferation and to cause cell apoptosis [62]. The chemotherapeutic drugs like doxorubicin (DOX), cisplatin, paclitaxel (PTX), mitoxantrone (MTX), and 5-fluorouracil (5-FU) have been proved to inhibit growth rates of different cancer cells and to limit their metabolic functions. However, the associated unwanted complications with these drugs due to their cytotoxicity towards normal/healthy cells have limited the application of chemotherapy in cancer therapy. Hence, nanocarrier-based drug delivery system was introduced as an effective approach to alleviate this limitation, with the aim to deliver chemotherapeutic drugs with minimum side effects. Indeed, many nanomaterials—such as polymeric nanoparticles, liposomes, micelles, and metal nanoparticles—have been employed for this purpose to deliver different chemotherapeutic drugs due to their unique characteristics unattainable through free drug administration. Recently, researchers are turning to development of rGO-based drug delivery platforms for carrying large amount of chemotherapeutic drugs with large surface area, as well as the pH-responsive drug release behavior offered by rGO.

Ma and co-workers used a green approach to convert GO to rGO using riboflavin as a reducing agent and the resulting riboflavin-rGO nanocarrier was used for DOX loading through π–π interaction [63]. The results suggested that rGO exhibits high DOX loading, good stability, and pH-sensitive sustained drug release, which is evident from the effective cytotoxicity against MCF-7 and A549 cancer cells in vitro [64]. In another study, Wei et al. used rGO-C_6_H_4_-COOH for DOX loading, followed by modifying with PEI to enhance water solubility and conjugating with folic acid (FA) for targeted drug delivery. Due to specific targeting of FA to CBRH7919 cancer cells as well as pH-responsive drug release after endocytosis, the conjugation of DOX with rGO-PEI-FA could arrest cancer cells in the G2 phase and lead to cell apoptosis. There are plenty of other examples for rGO-based targeted delivery of DOX. Daysi et al. also used FA-functionalized nanocomposite consisting of chemically-reduced rGO and manganese-doped zinc sulfide quantum dots (FA-rGO/ZnS:Mn) for targeted delivery of DOX [65]. The dispersion stability, DOX loading and release efficiency, internalization, and biocompatibility of FA-rGO/ZnS:Mn resulted in excellent anti-cancer efficiency against breast cancer cells. Moreover, FA functionalization improved the selectivity of this drug delivery platform for specific targeting of folate receptor molecules overexpressed on cancer cell surface. Taken together, the FA-rGO/ZnS:Mn was suggested to be an excellent theranostic nanocomposite for breast cancer treatment. Similarly, Miao et al. explained the application of DOX-loaded cholesteryl hyaluronic acid (CHA)-coated rGO nanosheets (CHA-rGO) for the treatment of CD44-overexpressing tumors [66]. As a primary ligand of CD44, HA increases the stability and safety of rGO by enhancing the tumor-targeted distribution of DOX to CD44-overexpressing cancer cells, thereby providing better drug accumulation and substantial reduction of tumor volume from in vivo study in nude mice. He and co-workers used rGO capped by alkyl-grafted mesoporous silica (MSN-C_18_) as a carrier of DOX for chemotherapy [67]. The in vitro cell viability assay performed using SMMC-7721 cancer cells showed enhanced DOX release upon near NIR light exposure, which led to higher cytotoxicity toward cancer cells, indicating its potential use as a nanocarrier for controlled drug release.

The application of rGO in chemotherapy is not only limited to targeted delivery of DOX, as other drugs or natural compounds with anti-cancer activity were reported to be delivered through rGO. Chen et al. used methoxypolyethylene glycol amine (mPEG-NH_2_) for one-step green reduction and PEGylation of GO to synthesize rGO/PEG [68]. The rGO/PEG showed excellent water stability and two-fold increase of resveratrol loading over GO/PEG via hydrophobic interactions and π–π stacking. From in vitro experiments, NIR laser irradiation (808 nm) could enhance resveratrol release from rGO/PEG to increase the cytotoxicity against 4T1 murine breast cancer cells by lowering cell viability and inducing cell apoptosis. In animal models with subcutaneously implanted cancer cells, resveratrol-loaded rGO/PEG injected intratumorally to tumor-bearing nude mice also significantly suppress tumor growth under photothermally controlled drug delivery. In another study, rGO synthesized through reduction by Euphorbia milii plant extract was used as a carrier of the chemotherapeutic drug paclitaxel for cancer treatment [69]. The drug-loaded rGO showed high cytotoxicity toward human lung cancer cell line (A549) for potential chemotherapy of lung carcinoma.

The chemotherapy using dual drugs may be a better approach to kill cancer cells in the metastatic stage as the synergistic effect offered by dual drugs may introduce more anti-proliferative effect to cause cancer cell death. Muthoosamy et al. developed amphiphilic polymer PF-127 functionalized rGO for co-loading of anti-cancer drugs paclitaxel and curcumin on the surface through π–π interactions [70]. The drug-loaded composite showed synergistic anti-tumor efficacy towards both A549 lung cancer cells and MDA-MB-231 breast cancer cells. However, loading of an aromatic hydrophobic drug like paclitaxel on the surface of a hydrophobic carrier like rGO by π–π stacking and hydrophobic–hydrophobic interactions is challenging as hydrophobic carriers are not stable in physiological solutions. To address this problem, Hashemi et al. introduced a rGO-based nanocarrier with high paclitaxel loading capacity through functionalization and stabilization with R9 peptides, where pristine rGO sheets were found to be unstable in aqueous solutions and aggregated to decrease the surface area available for drug loading [71]. In a different study, Dhanavel et al. developed dual drug-encapsulated chitosan/rGO nanocomposite by entrapping 5-fluorouracil (5-FU) and curcumin in chitosan/sodium tripolyphosphate gel in the presence of rGO nanosheet for dual drug delivery to HT-29 colon cancer cells [72]. The synergistic cytotoxicity was observed for dual drug-loaded nanocomposite to inhibit the growth of HT-29 colon cancer cells compared with single drug therapy.

Considering the biological aspects, researchers have developed composites of rGO with nanoparticles or polymers for cancer treatment. Among these, combination of rGO with gold (Au) nanoparticle has gained considerable attention. In one study, Sanad et al. prepared rGO–gold nanocomposites (rGO-Au) by incorporating Au nanoparticles inside the rGO matrix through in situ reduction with sodium borohydride, followed by loading 5-FU by pore capping [73]. The results obtained from cytotoxicity assay determined by the reduced half maximal inhibitory concentration (IC_50_) using MTT assay in addition to enhanced cell apoptosis from flow cytometry analysis suggested the nanocomposite can enhance targeted delivery of 5-FU as well as cytotoxicity to MCF-7 breast cancer cells. In a similar study, Jafarizad et al. prepared Au nanoparticle-loaded rGO as a covalent drug delivery system for pH-dependent release of mitoxantrone (MTX) [74]. For polymer coating, Ryu and co-workers prepared PEI-rGO nanocarrier for pH-responsive delivery of DOX to Hela and A549 cancer cell lines [75]. The drug-loaded nanocomposite was further coated with pH-responsive charge-conversional polymer polyethyleneimine-poly-_L_-lysine-poly-_L_-glutamic acid (PKE) to endow charge-conversional property and serum stability to PEI-rGO-based drug delivery system. They found that DOX-loaded PEI-rGO after PKE coating released more DOX under low pH lysosomal condition and showed enhanced anticancer activity in HeLa and A549 cancer cells. Considering the side effects associated with chemotherapy, SreeHarsha et al. prepared hybrid nanoparticle by coating rGO with chitosan and stabilized with tripolyphosphate to produce stabilized nanocomposite for delivery of DOX to PC-3 cancer cells [76]. The sustained DOX release observed under photothermal conditions endowed this nanocarrier with improved efficacy in treating prostate cancer.

### 3.2. Photothermal Therapy (PTT) and/or Photodynamic Therapy (PDT)

Photothermal therapy (PTT) involves local temperature rise after exposing a photothermal agent to electromagnetic radiation such as visible or NIR light, which up converting light energy into heat, can induce death of cancer cells [77]. Many nanomaterials are effective photothermal agents in causing cancer cell apoptosis/necrosis with local hyperthermia from NIR laser exposure [78]. Graphene-based materials are good photothermal agents used for PTT considering its multifunctionality [79]. Moreover, the combination of graphene-based materials with inorganic particles, like iron oxide and gold nanoparticles, can further enhance the photothermal effect and lead to higher cancer cell death rate during PTT [80,81,82,83]. On the other hand, photodynamic therapy (PDT) is another form of phototherapy involving light and a photosensitizer (PS), which when used together with oxygen, can produce molecular oxygen or reactive oxygen species to elicit cancer cell death [84,85]. In one study, GO was fond to act both as a photothermal agent for PTT and a photosensitizer for PDT, making it an excellent candidate for synergistic phototherapy [86].

Robison et al. pioneered the use of rGO for PTT, who showed nano-sized rGO produced by chemical reduction of GO has six-fold higher NIR absorption rate than GO, endorsing its preferred use over GO as a photothermal agent [87]. The modification with targeting peptide bearing the Arg-Gly-Asp (RGD) motif further provided rGO with selective cellular uptake ability by U87MG glioma cancer cells, indicating rGO is a multi-functional photothermal agent. Their results also provided strong evidence that nano-sized rGO is highly effective as a photothermal agent when compared to other carbon-based nanomaterials as well as inorganic nanoparticles like gold or iron oxide. Similarly, Shim et al. modified rGO with clostridium perfringens enterotoxin peptide-linked chlorin e6 (Ce6) as a dual photodynamic and photothermal cancer therapeutic platform [88]. The intracellular uptake studies performed on U87 glioblastoma cells confirmed the ligand-mediated cellular uptake. The combined therapy using 660 nm light source for the PDT agent (Ce6) and 808 nm for rGO showed enhanced targeted dual phototherapy. In another study, He and co-workers synthesised palladium nanoflowers-decorated rGO (rGO/PdNFs) and explored its versatile applications in catalysts, sensor, and PTT [89]. The modification of rGO with PdNFs increased its photothermal conversion due to enhanced absorption in the NIR window. Both in vitro study using HeLa cells and in vivo animal experiments performed in tumor-bearing mice model indicates rGO/PdNFs could result in effective photothermal antitumor efficacy. The study using alanine-grafted rGO as a photothermal platform for cancer therapy confirmed that PTT using 808 nm laser irradiation produced 89% and 33% higher photothermal effect compared to GO and rGO, respectively. The conjugation of alanine to GO via π–π interactions reduced GO to rGO, not only increased the 808 nm absorbance but also acted as a targeting ligand to kill U87MG cancer cells selectively [90]. 

The photothermal applications rGO were further explored in combination with other nanoparticles and photosensitizers in synergistic cancer therapy. Otari et al. performed one-step reduction of GO to rGO and decoration of rGO with Au nanoparticles and thermostable antimicrobial nisin peptides to synthesize NAu-rGO [91]. After treating MCF-7 breast cancer cells with the nanocomposite followed by 800-nm diode laser (0.5 W/cm^2^) treatment for 5 min, 80% cell growth inhibition was found. Similarly, Zhang et al. attached polyethylene glycol (PEG) modified Ru (II) complex (Ru-PEG) to rGO surface by hydrophobic π–π interaction, and applied the nanocomposite as a photothermal agent and a photosensitizer for PTT/PDT [92]. The A549 lung cancer cells treated with rGO-Ru-PEG and sequentially exposed to of 808 nm (for PTT) and 450 nm (for PDT) wavelength light source resulted in enhanced cytotoxicity due to combined phototherapeutic effects. The rapid reduction in relative tumor volume observed from animal experiments also confirmed the synergistic effect of dual phototherapy.

For localized combination cancer therapy, Chang and co-workers developed rGO/AE/AuNPs hydrogel containing rGO and Au nanoparticles, by using amaranth extract (AE) both as a reducing agent and a precursor, which crosslinked upon 660 nm laser exposure to form a composite hydrogel [93]. Both Au nanoparticles and rGO acted as photothermal agents while the chlorophyll derivatives in AE acted as a photosensitizer to accelerate the generation of cytotoxic singlet oxygen. Upon hydrogel formation on the surface of HeLa cancer cells in situ, the composite hydrogel was used as a combined PTT/PDT platform by repeated irradiation with 808 nm laser in multiple antitumor therapies. In another study, a PTT/PDT reagent was synthesized by conjugating tetrakis(4-carboxyphenyl) porphyrin (TCPP) to rGO–PEI, which was formed based on carboxylic acid functionalized rGO for combination PTT/PDT therapy [94]. The rGO–PEI–TCPP composite showed excellent stability in different biological solutions. The results obtained from studies with CBRH7919 cancer cells indicates induced cell apoptosis upon laser irradiation, due to the combined photothermal and photodynamic effects with the production of heat and singlet oxygen. The increase in temperature upon exposing a photothermal agent to laser light may cause side effects, and hence determining the optimum concentration of PTT agent or performing experiment at lower laser power is important in phototherapy. Jafarirad and team developed a non-invasive strategy for low-level laser induced cancer therapy. The hybrid nanocomposites (ZnO/rGO, Ag-ZnO/rGO, and Nd-ZnO/rGO) synthesized by green synthesis methods were further optimized for concentration in anti-tumor study in vitro using MCF-7 cancer cells [95]. The results confirmed a low concentration (12.5 µg/mL) of hybrid together with low irradiation doses (8–32 J/cm^2^) could lead to higher cell death.

In conventional PDT, the unfavourable bioavailability, low absorption band and limitations in tissue oxygenation are considered as possible limitations. To overcome these limitations, Kapri et al. fabricated a ∼5 nm thick MoS_2_ nanoplatelet and integrated them with n-type nitrogen doped rGO for PDT [96]. The p-MoS_2_/n-rGO-MnO_2_-PEG composite was prepared by modifying the nanosheet with poly(ethylene glycol) (PEG) to improve biocompatibility and colloidal stability in physiological solution, which was further surface decorated with MnO_2_ to overcome the hypoxic conditions prevalent in tumor microenvironment, by increasing intracellular O_2_ after reaction of MnO_2_ with endogenous H_2_O_2_ in cancer cells. The nanosheet reveals increased apoptosis under NIR light irradiation by alleviating hypoxia and enhances the efficacy of PDT on HeLa cells in vitro. It is difficult to selectively kill cancer cells during PTT as normal cells are also simultaneously affected by the photothermal effect, which is the most common disadvantage associated with phototherapy. To solve this problem, an interesting design based on rGO was demonstrated by synthesizing water dispersible Cu_2_O nanocrystal-rGO nanocomposites. In contrast to the highly efficient killing of both normal and cancer cells initiated by the photothermal effect under NIR irradiation, the photocatalytic effect of this nanomaterial results in selective killing of cancer cells in contrast to unselective cell-killing under NIR light [97]. This was demonstrated from the cytotoxicity assay performed on A549, HK-2 and MDA-MB-231 cancer cell lines in vitro.

The efficacy of rGO-based PDT/PTT could be upregulated by conjugating rGO with a ligand molecule to actively targeting cancer cells. Jiang et al. used hyaluronic acid (HA) as a targeting ligand to specifically deliver a photosensitizer Ce6 to CD44 over-expressing cancer cells for PDT with NIR irradiation [98]. The nanoplatform was prepared by dopamine-reduced rGO sheet and coated with mesoporous silica to load Ce6 as well as HA. The combination of photothermal conversion and controllable Ce6 release after NIR irradiation confers the nanoplatform with enhanced singlet oxygen generation that could lead to more significant destruction of targeted cancer cells. Lima-Sousa and co-workers also functionalized rGO with HA-grafted poly-maleic anhydride-*alt*-1-octadecene (HA-g-PMAO) for targeted PTT [99]. In vitro studies confirmed internalization by CD44 overexpressing MCF-7 cells as an on-demand PTT platform to elicit cancer cell ablation for potential targeted cancer therapy. Taking advantage of the fact that polyphenol compounds in green tea (GT)-reduced rGO can act as targeting ligand, targeted delivery of the nanocomposite resulted in 20% higher photothermal destruction of the high metastatic SW48 cancer cells than that of the low metastatic HT29 cells [100]. Although the exact mechanism is still under investigation, the attachment of polyphenol-modified rGO to cancer cell surface could be confirmed from flow cytometry studies for photothermal destruction of colon cancer cell line at 0.3 mg/mL rGO and 0.25 W/cm^2^ NIR laser power density. To use heparin sulphate proteoglypican-3 (GPC3) as a targeting ligand for hepatocellular carcinoma, Liu et al. conjugated biotinylated GPC3 antibody to rGO (rGO-GPC3) and bind avidinylated nanobubbles to rGO-GPC3 using the biotin-avidin bioaffinity system for PTT. Using ultrasound-targeted nanobubble destruction, the local concentration of rGO around HepG2 cell line could be increased for photothermal ablation and PTT of hepatocellular carcinoma under 808 nm NIR irradiation [101].

Indocyanine green (ICG), a NIR dye approved by the U.S. Food and Drug Administration (FDA), can combine with rGO to promote the NIR absorption ability of rGO and enhance the PTT efficacy. A novel nanoagent using ICG-loaded polydopamine (PDA)-reduced graphene oxide nanocomposites (PDA-rGO) was found to be loaded with a large amount of ICG molecules for exhibiting stronger photothermal effect and amplify the PTT efficacy for cancer theranostics [102]. After photoacoustic imaging-guided PTT treatments using 808 nm NIR laser at 0.6 W/cm^2^ for 5 min, the tumors in orthotopic 4T1 breast cancer mice model were completely eradicated with no observable treatment toxicity. Also using ICG, Sharker et al. designed a pH-responsive, NIR-sensitive rGO-based nanocomposite (ICG-CPPDN/rGO), by ionic complexation of ICG with CPPDN/rGO, for local destruction of cancer cells with minimal invasiveness to surrounding normal cells [103]. The nanocomposites showed pH-dependent photothermal effect from pH 5.0 to 7.4 due to the pH response relief and quenching effects of ICG on rGO sheet, which leads to photo-thermolysis as the pH was changed from 5.0 to 7.4 in vitro. Due to acidic tumor microenvironment, the nanocomposite showed improved photothermal destruction of MDA-MB-231 cancer cells both in vitro and in vivo compared to free ICG upon local NIR laser treatment.

### 3.3. Gene Therapy

Inhibiting gene expression by promoting site specific cleavage of target messenger RNA, small interfering RNA (siRNA) regulates the expression of genes by RNA interference (RNAi) and represents one of the promising developments in cancer therapy [104]. Considering the limitations of delivery of naked siRNA, which include endosomal escape, rapid excretion, low stability in blood serum, non-specific accumulation in tissues, siRNAs were usually delivered by loading to nanoparticles [105]. The use nanoparticles as nano-vehicles for siRNA delivery also offers the possibility of targeted gene therapy for more effective cancer treatment outcomes [106]. Various nano-sized particulate systems, including silica and silicon-based nanoparticles, metal and metal oxide nanoparticles, carbon nanotube, graphene, dendrimer, polymers, cyclodextrin, liposome, and semiconductor nanocrystals have been developed for systematic delivery of siRNA [107]. The cationic PEI-rGO nanoparticles after reducing and modifying GO with PEI have gained particular attention in rGO-based cancer gene therapy, since PEI is widely used for non-viral transfection and offers advantages over other polycations with high endosomolytic activity and strong DNA compaction ability [108,109].

As low molecular weight PEI was proved to exhibit less cytotoxicity, Chau et al. studied the covalent functionalization of GO with triethyleneglycoldiamine or 800 Da molecular weight PEI via the epoxy ring opening reaction. The data obtained using gel electrophoresis confirmed that PEI-rGO was more efficient in complexing with siRNA to offer higher complexing capacity, making it an excellent candidate for gene silencing applications [110]. In a separate study, rGO was modified with low molecular weight branched polyethyleneimine (BPEI) via polyethylene glycol (PEG) spacer (PEG-BPEI-rGO) as a nano-vehicle for photothermally controlled gene delivery [111,112]. The nanocomposite formed stable nano-sized complex with plasmid DNA to offer high gene transfection efficiency for experiments performed with PC-3 and NIH/3T3 cell lines without significant cytotoxicity. Most importantly, PEG-BPEI-rGO demonstrated enhanced gene transfection efficiency upon NIR irradiation. After investigating with a proton sponge effect inhibitor Bafilomycin A1, the enhancement of gene transfer was found to be associated with accelerated endosomal escape of the nanocomposite, with the photothermal effect of rGO.

### 3.4. Chemotherapy/Phototherapy

**Figure 3:**
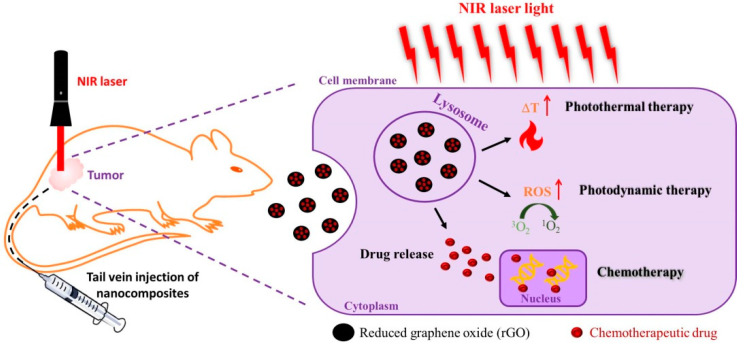
A schematic diagram showing the treatment of subcutaneously implanted cancer cells by combined chemotherapy/phototherapy using reduced graphene oxide (rGO).

As chemotherapy and phototherapy have produced promising outcomes in cancer therapy, researchers started combining these therapeutic modalities for synergistic cancer treatment [113]. Specifically, considering the high loading efficiency of chemotherapeutic drugs and the unique NIR laser-responsive characteristics for PTT/PDT, graphene-based nanomaterials have received attention for chemo-phototherapy [114]. By intravenous delivery of rGO-based nanocomposites, the combination of chemotherapy with phototherapy (PTT and/or PDT) also demonstrated promising anti-cancer efficacy with subcutaneously implanted cancer cells in vivo, as depicted schematically in Figure 3 [115,116].

Oz and co-workers functionalized rGO by surface anchoring maleimide-containing catechol (dopa-MAL) through noncovalent interaction. Using thiol–maleimide chemistry, they modify rGO with cyclic peptide c(RGDfC) as a targeting ligand for targeted delivery of DOX to cancer cells. The in vitro studies performed with MDA-MB-231 cell line indicated that DOX loaded rGO/dopa-MAL-c(RGDFC) was more effective than free DOX in killing the cancer cells after exposure to 980 nm laser irradiation (2 W/cm^2^) for 10 min [117]. This was attributed to the enhancement of chemotherapy with PTT from the endocytosed nanocomposite upon NIR laser exposure for targeted synergistic cancer cell killing. Surface functionalization of rGO for enhancing the hydrophilicity is a strategy for effective drug delivery. Hu et al. used folic acid modified dextran-g-octadecanoic acid to decorate rGO surface with hydrophilic dextran moiety through octadecanoic acid hydrophobic anchoring and with folic acid for enhanced intracellular uptake by cancer cells. The nanocomposite was loaded with anticancer drug DOX for chemotherapy/phototherapy where in vitro analysis performed with HeLa cells at endosomal acidic environment (pH 5.3) confirms increased DOX release upon weakening of noncovalent binding between DOX and rGO. Compared with single mode chemotherapy, the combination of local chemotherapy with external NIR-induced PTT demonstrated dual therapy and offered higher therapeutic efficacy [118]. The study confirmed that by eliciting higher cytotoxicity toward cancer cells through the photothermal response of rGO under NIR irradiation, the concentration of DOX needed for chemotherapy could be significantly reduced to minimize the potential side effect of chemo drugs. Similarly, Hu and co-workers also delivered DOX using dextran-reduced rGO through direct conjugation of dextran on rGO surface by hydrogen bonds. This was followed by self-assembly to form rGO/Dex nanoparticles. After conjugating with RGD peptide for recognition by α_v_β_3_ integrin on cancer cell surface to enhance intracellular uptake, the in vitro chemo-phototherapy performed with external NIR laser on B16F10 cell line resulted in higher anti-cancer efficacy [119].

Considered as one of the most widely used chemotherapeutic drugs, DOX has been loaded to rGO synthesized with various reducing agents. For this purpose, functionalized rGO with bovine serum albumin (BSA) as a reduced agent was used as a carrier of DOX [57]. Brain tumor cells (U87MG) treated with BSA-rGO revealed that combination of photo-chemo treatment enhanced the treatment efficacy as compared to single mode phototherapy using BSA-rGO or DOX (chemotherapy). Similarly, Zaharie-Butucel and team reduced GO using chitosan to combine PTT and PDT, followed by using chitosan-rGO as a carrier for DOX in synergetic therapy of colon cancer [49]. Targeted delivery of DOX using polydopamine-functionalized rGO (pRGO) is another example in this category [120]. pRGO modified with CD44 targeting ligand hyaluronic acid (HA) was used in combination with DOX-loaded mesoporous silica (MS) (pRGO@MS-HA) to generate both pH and NIR-triggered DOX release from the multifunctional nanosystems, showing excellent combined effect in multimodal cancer therapy. In another example, Hao et al. used tea polyphenol to produce rGO as a nanocarrier for delivery of DOX [121]. After exposure to 808 nm NIR laser irradiation at 3 W/cm^2^ for 5 min, combined chemo-PTT can enhance cytotoxicity of DOX toward human tongue squamous cancer cells, CAL27.

To improve photothermal properties, Ma et al. added gold (Au) clusters to rGO surface by electrostatic interaction. Afterward, rGO was functionalized with 3-(3-phenylureido) propanoic acid–polyethylene glycol (PPEG) via π–π bond interaction for improving the biocompatibility. Using DOX for chemotherapy, rGO/Au/PPEG elicited effective phototherapy/chemotherapy effect on HeLa cell line [122]. In a separate study, Yang et al. introduced Au nanorods as well as hydroxyapatite to rGO surface (RGO/AuNR/HA) for delivery of the anticancer drug 5-fluorouracil (5-FU). The nanocomposite was designed for synergistic dual therapy, in which hydroxyapatite was used to enhance 5-FU loading, while RGO and Au nanorods (AuNR) offer enhanced photothermal effect under NIR laser irradiation [123]. Due to sequential drug release with the pH-sensitive drug release behavior of hydroxyapatite in the first stage and photothermal conversion from RGO/AuNR after NIR laser irradiation in the second stage, the designed nanocomposite exhibits greater antitumor activity from the chemo-photo effect. A hybrid with ultra-small plasmonic gold nanorods vesicles (rGO-AuNRVe) loaded on the surface of rGO was shown be to be endowed with amplified photothermal effect. This hybrid could provide a high loading capacity of DOX, provided by the cavity of the vesicle and the large surface area of rGO. Furthermore, the release of DOX was sequential with DOX release first from the vesicular cavity under NIR photothermal heating and followed by release from rGO surface induced by the intracellular acidic environment [124]. Intravenous injection of rGO-AuNRVe-DOX followed by low power 808 nm NIR laser irradiation (0.25 W/cm^2^) leads to effective inhibition of tumor growth of subcutaneously implanted U87MG human glioblastoma cells in nude mice from combinatory chemo- and photothermal therapies.

Of course, the application of rGO in chemo-phototherapy is not limited to DOX as there are reports employing different chemotherapeutic drugs in combination with rGO to enhance the cytotoxicity of the chemo drug through combination with PTT. In one study, a nanocookie prepared by coating amorphous carbon on a mesoporous silica support (PSS) and self-assembled on rGO nanosheet, was used as a photo-responsive drug carrier for delivery of a hydrophobic anticancer drug camptothecin (CPT). Other than providing a large payload of CPT, this nanocomposite provided a burst-like drug release and intense photothermal effect upon NIR exposure [125]. The tumor volume change observed from MDA-MB231 tumor-bearing nude mice confirmed chemo-phototherapy due to synergistic photothermal and chemo therapeutic effects (nanocookie–CPT + NIR) was more effective than chemotherapy alone (nanocookie-CPT) or photothermal effect alone (nanocookie + NIR). In a separate study, Vinothini at el. developed magnetic iron oxide nanoparticles functionalized rGO for loading CPT. Furthermore, a photosensitizer 4-hydroxycoumarin (4-HC) was bound to rGO via allyl amine (AA) linker. The cytotoxicity study using MCF-7 human breast cancer cells upon 365 nm laser irradiation at 20 mW/cm^2^ for 3 min indicated CPT-loaded MrGO-AA-g-4-HC could produce reactive oxygen species (ROS) for killing of MCF-7 cancer cells for PDT. Unfortunately, with the limited penetration depth of light source in the visible wavelength range (365 nm) for inducing PDT effect of 4-HC, the in vivo results only demonstrated significant tumor growth suppression for CPT-loaded MrGO-AA-g-4-HC without laser treatment [126].

### 3.5. Photothermal Therapy/Immunotherapy

Immunotherapy, which kills cancer cells by improving immunity, is a new approach for cancer therapy. The success of immunotherapy is determined by two main tools, checkpoint inhibitors (CPIs) and chimeric antigen receptor (CAR) T cells [127,128]. Although immunotherapies have achieved promising results against metastatic cancers, traditional immunotherapies are often expensive and can have toxic side effects. During the process of PTT, the heat generated by the photothermal agent not only ablates the tumor but also produces tumor-associated antigens by causing immunogenic cell death, which can lead to antitumor immunity in the body. Hence, the combination of PTT and immunotherapy (photo-immunotherapy) has shown great promise in cancer therapy recently [129,130]. Although many nanoparticles have been used for photo-immunotherapy, rGO stands out as one of the best choice among them [131]. Wang et al. prepared a nanocomposite consisting of PEGylated rGO hybridized with iron oxide nanoparticles through electrostatic interaction for photothermal-immunotherapy of metastatic cancer. This nanocomposite was an excellent photothermal agent for direct killing of cancer cells by PTT, which also stimulated immune responses by triggering the maturation of dendritic cells as well as the secretion of cytokines to cause immunogenic cell death of tumor cells [131]. In vivo antitumor studies revealed the nanocomposite to be an excellent photothermal agent for PTT when exposed to NIR laser to destroy primary tumor effectively. After NIR laser treatment of 4T1 orthotopic mouse breast tumor, the intratumorally injected nanocomposites could significantly increase the survival time of tumor-bearing mice by eliciting strong antitumor immunological response of the treated animal.

Yan et al. also combined immunotherapy with PTT with folic acid as a targeting ligand by conjugating indoleamine-2,3-dioxygenase (IDO) inhibitor to rGO to induce IDO inhibition and programmed cell death-ligand 1 (PD-L1) blockade for synergistic antitumor immunity. After laser irradiation, the nanocomposite can directly kill tumor cells due to PTT and trigger antitumor immune response synergistically by IDO inhibition as well as PD-L1 blockade in CT26 colon cancer cells. By combining PTT, IDO inhibition, and PD-L1 blockade, the growth of irradiated tumor in distant sites without PTT treatment can be effectively inhibited by targeting multiple antitumor immune pathways to induce synergistic antitumor immunity [132].

## 4. Conclusions and Outlook

Due to improved photothermal response by absorbing light in the NIR range and the potential for high loading of chemotherapeutic drugs, photosensitizers and siRNA, rGO synthesized by means of various reducing agents is well suited for applications in single or multi-mode cancer therapy. Based on the reducing agent used for rGO synthesis, and the moieties conjugated with it, rGO-based nanocomposite is endowed with triggered drug release capability after intracellular uptake, by pH change or hyperthermia. This temperature-dependent and pH-responsive drug release, when combined with PTT and/or PDT, can lead to pronounced cytotoxicity from in vitro and in vivo studies performed with various cancer cells. rGO can also act as a good vehicle for gene delivery after modification/conjugation with cationic polymers, especially PEI, which can act alone for RNAi or combined with rGO-induced PTT for combination therapy involving immunotherapy. Overall, this review concludes that rGO is a promising and versatile tool after functionalization for cancer therapy, especially in combination cancer therapy such as PTT/PDT, chemotherapy/phototherapy and photothermal therapy/immunotherapy to elicit synergistic anti-tumor efficacy. Undoubtedly, despite remarkable therapeutic efficacy demonstrated from combination cancer therapy using rGO, rationally combining the therapeutic modalities into rGO-based platform for ‘smart’ drug delivery will be desirable. In addition, for successful cancer therapy, the designed rGO-based nanocomposites should preferably be endowed with both therapeutic and diagnostic functions for precision nanomedicine. Additionally, the function of combined cancer therapeutics offered by rGO may be required to be programmed for realizing the synergistic effects. Moreover, it would be helpful to develop better PDT/PTT cancer therapeutics using rGO, which can alleviate the limit of penetration depth of NIR laser for effective eradication of tumors located deep in the body.

## Figures and Tables

**Table 1:** Reducing agents for producing reduced graphene oxide (rGO) from graphene oxide (GO).

Reducing Agent	Characterisitics	Applications	Reference
Vitamin C	Natural compound; non-toxic; mild reaction temperature; environment friendly byproducts; avoid introducing heteroatoms; reaction in aqueous or organic solution	Embedded in chitosan hydrogel for bone tissue engineering; functionalized with antimicrobial peptide for antibacterial activity	[37,38,39]
Hydrazine hydrate	Explosive; toxic; large scale production; low cost	Improve electrical conductivity; embedded in polyacrylic acid nanofiber mats for controlled release of antibiotics	[41,42,43,44]
Resveratrol	Natural phenolic compound; anti-oxidant; stabilizer; biocompatibility; solubility; green synthesis	Produce marked changes in cellular morphology and reduce cell viability of cancer cells for cancer therapy	[45]
Chitosan	Biocompatible; biodegradable; reduction at body temperature; biological and medicinal applications	Reversible change of dispersion/aggregation state with pH; pH-sensitive release of drug; loading with drug and photosensitizer for cancer chemotherapy/phototherapy	[48,49]
Polyethylenimine	Surface modifier; one-step hydrothermal reduction; high cargo loading; prevent agglomeration	Improved gas barrier property in composite films; in hemin-bovine serum albumin composite as peroxidase mimetics; gene delivery; increase strength of nylon composites	[50,51,52]
Sodium borohydride	Efficient; ambient conditions; reaction in aqueous solution	Decrease electrical resistance; enhance electrical conductivity	[53,54]
Bovine serum albumin	Biocompatible; stabilizer; binding by adhesion to surface; metal particle-binding platform; cell adhesive	For cancer chemo-photothermal therapy; adsorption and assembly of metal particles; create protein–metal nanocluster for detecting trypsin	[57,58]
Gree tea polyphenols	Biocompatible, biodegradable; green synthesis; good dispersion in both aqueous and organic solutions; non-toxic	Enhance thermal conductivity in chitosan polymer composites; deposite onto electrode for detection of sunset yellow in foods; reduce cytotoxicity of GO	[59,60,61]

**Table 2:** Summary of functionalized rGO-based nanocomposites used for cancer therapy.

Nanocarrier	Functionalization Agent	Cancer Cell Line	Type of Study	Reference
**Chemotherapy**				
Riboflavin-rGO	DOX, riboflavin	MCF-7, A549	In vitro	[63]
rGO-PEI-FA	DOX, folic acid (FA)	CBRH7919	In vitro	[64]
FA-rGO/ZnS:Mn	DOX, folic acid (FA), Mn-doped ZnS quantum dots	MDA-MB-231	In vitro	[65]
CHA-rGO	DOX, cholesteryl hyaluronic acid (CHA)	KB	In vitro, in vivo	[66]
PEG-BPEI-rGO	DOX, branched polyethylenimine (BPEI), polyethylene glycol (PEG)	PC-3	In vitro	[112]
NrGO/PEG	Resveratrol, PEG	4T1	In vitro, in vivo	[68]
MSN-C_18_-rGO	DOX, mesoporous silica grafted with alkyl chains (MSN-C_18_)	SMMC-7721	In vitro	[67]
GP	PF-127 polymer, curcumin, paclitaxel	A549, MDA-MB-231	In vitro	[70]
CS/rGO	Chitosan (CS), 5-FU, curcumin	HT-29	In vitro	[72]
R9-rGO	R9 peptide, paclitaxel	HeLa, MCF-7	In vitro	[71]
rGO-Au	5-FU, gold (Au)	MCF-7	In vitro	[73]
MPA-AuNPs/rGO	MTX, SMTX-gold nanoparticles (AuNPs)	MCF-7	In vitro	[74]
PK_5_E_7_(PEI-rGO)	DOX, PK_5_E_7_ polymer, PEI	Hela, A549	In vitro	[75]
rGOD-hNP	DOX, chitosan	PC-3	In vitro	[76]
RGO	Leaf extract, paclitaxel	A549	In vitro	[69]
**Photothermal and/or Photodynamic Therapy**				
rGO-RGD	RGD peptide	U87MG	In vitro	[87]
CPC/rGO	Chlorin (Ce6), claudin 4-binding peptide	U87, HeLa	In vitro	[88]
rGO/PdNFs	Palladium nanoflowers (PdNFs)	HeLa	In vitro, in vivo	[89]
Ag(Nd)-ZnO/rGO	Ag(Nd)/ZnO	MCF-7	In vitro	[95]
ARGO	Alanine	U87MG	In vitro	[90]
ICG-CPPDN/rGO	Catechol, PPDN polymer, ICG	MDA-MB-231	In vitro, in vivo	[103]
rGO/AE/AuNPs	Amaranth extract (AE), gold nanoparticles (AuNPs)	HeLa	In vitro	[93]
rGO-Ru-PEG	PEG, Ru(II)	A549	In vitro, in vivo	[92]
NAu-rGO	Nisin peptides, gold nanoparticles (AuNPs)	MCF-7, HeLa	In vitro	[91]
p-MoS_2_/n-rGO-MnO_2_-PEG	p-type molybdenum sulfide (p-MoS_2_), MnO_2_, PEG	HeLa, HEK293	In vitro	[96]
Cu_2_O-rGO	Cu_2_O	HK-2, MDA-MB-231, A549	In vitro	[97]
rGO-PEI-TCPP	Polyethyleneimine (PEI), tetrakis(4-carboxyphenyl) porphyrin (TCPP)	CBRH7919	In vitro	[94]
rGO-PDA@MS/HA	Mesoporous silica (MS), hyaluronic acid (HA), polydopamine (PDA), Ce6	HT-29, HCT-116	In vitro	[98]
rGO/HA-g-PMAO	Hyaluronic acid (HA) grafted PMAO	MCF-7, NHDF	In vitro	[99]
GT-rGO	Green tea	SW48, HT29	In vitro	[100]
NBs-GPC3-rGO	GPC3 antibody, nanobubbles	HepG2	In vitro	[101]
ICG-PDA-rGO	ICG, polydopamine	4T1	In vitro, in vivo	[102]
**Gene Therapy**				
rGO-PEI	PEI, siRNA	None	None	[110]
PEG-BPEI-rGO	Low molecular-weight branched polyethylenimine (BPEI)	PC-3	In vitro	[111]
**Chemotherapy/Phototherapy**				
rGO/dopa-MAL-c(RGDfC)	Catechol, DOX, c(RGDfC) peptide	HeLa, MDA-MB-231	In vitro	[117]
rGO/C18D	DOX, octadecanic acid conjugated on dextran (C18D)	HeLa	In vitro	[118]
rGO@PSS	Camptothecin (CPT), mesoporous silica	MDA-MB-231	In vitro, in vivo	[125]
rGO/Dex	DOX, dextran, RGD peptide	B16F10	In vitro	[119]
BSA-rGO	DOX, bovine erum albumin (BSA)	U87MG	In vitro	[57]
rGO/Au/PPEG	DOX, 3-(3-phenylureido) propanoic acid (PPA)-PEG (PPEG), Au	HeLa	In vitro	[122]
Chit-rGO-IR-820	DOX, chitosan, IR-820	C26	In vitro	[49]
pRGO@MS-HA	DOX, hyaluronic acid (HA), mesoporous silica, polydopamine	HeLa	In vitro, in vivo	[120]
TPDL1-rGO	DOX, tea polyphenol, anti-PDL1 antibody	CAL-27, PDLCs	In vitro	[121]
MrGO-AA-g-4-HC	CPT, 4-hydroxycoumarin (4-HC), magnetic nanoparticles, camptothecin	MCF-7	In vitro, in vivo	[126]
rGO/AuNR/HAP	5-FU, gold nanorod (AuNR), hydroxyapatite	HeLa	In vitro	[123]
rGO-AuNRVe	DOX, gold nanorod vesicle	U87MG	In vitro, in vivo	[124]
**Photothermal Therapy/Immunotherapy**				
FNPs/rGO-PEG	Fe_3_O_4_ nanoparticles, PEG	4T1	In vitro, in vivo	[131]
PEG-rGO-FA-IDOi	IDO inhibitor (IDOi), folic acid, PEG	CT26	In vitro, in vivo	[132]
